# A Clinical Bridge between Family Caregivers and Older Adults: The Contribution of Patients’ Frailty and Optimism on Caregiver Burden

**DOI:** 10.3390/ijerph18073406

**Published:** 2021-03-25

**Authors:** Alberto Sardella, Vittorio Lenzo, Angela Alibrandi, Antonino Catalano, Francesco Corica, Maria C. Quattropani, Giorgio Basile

**Affiliations:** 1 Department of Clinical and Experimental Medicine, University of Messina, 98125 Messina, Italy; asardella@unime.it (A.S.); maria.quattropani@unime.it (M.C.Q.); 2 Department of Social and Educational Sciences of the Mediterranean Area, “Dante Alighieri” University for Foreigners of Reggio Calabria, 89125 Reggio Calabria, Italy; v.lenzo@unidarc.it; 3 Unit of Statistical and Mathematical Science, Department of Economics, University of Messina, 98123 Messina, Italy; angela.alibrandi@unime.it; 4 Department of Clinical and Experimental Medicine, School and Unit of Geriatrics, University of Messina, 98125 Messina, Italy; catalanoa@unime.it (A.C.); coricaf@unime.it (F.C.)

**Keywords:** caregiver burden, clinical psychology, dispositional optimism, family caregiver, frailty, older adults

## Abstract

The association between caregiver burden and the physical frailty of older adults has been the object of previous studies. The contribution of patients’ dispositional optimism on caregiver burden is a poorly investigated topic. The present study aimed at investigating whether older adults’ multidimensional frailty and optimism might contribute to the burden of their family caregivers. The Caregiver Burden Inventory was used to measure the care-related burden of caregivers. The multidimensional frailty status of each patient was evaluated by calculating a frailty index, and the revised Life Orientation Test was used to evaluate patients’ dispositional optimism. The study involved eighty family caregivers (mean age 64.28 ± 8.6) and eighty older patients (mean age 80.45 ± 7.13). Our results showed that higher frailty status and lower levels of optimism among patients were significantly associated with higher levels of overall burden and higher burden related to the restriction of personal time among caregivers. Patients’ frailty was additionally associated with caregivers’ greater feelings of failure, physical stress, role conflicts, and embarrassment. Understanding the close connection between patient-related factors and the burden of caregivers appears to be an actual challenge with significant clinical, social, and public health implications.

## 1. Introduction

Caregivers have an extremely delicate role, which is indeed accompanied by increasing social and public concern. Being a caregiver acquires a relevant importance in the context of several chronic medical conditions because of the different and various needs of patients [1,2,3,4].

In this context, older adults are a remarkably vulnerable population due to the progressive decline in physical and cognitive functions, as well as multimorbidity, which leads to a progressive loss of independence in daily life [5]. Family caregivers often take care of the needs of their older family members firsthand [6], which gives their role a great sense of responsibility and value [7]. However, the other side of the coin highlights the potential risk of family caregivers developing care-related burden, precisely due to the older subject’s health status [8,9]. In recent years, the construct of caregiver burden has also increasingly acquired a multifactorial dimension, which encompasses physical, psychological, emotional, social, and economic consequences for the caregivers [10].

Previous studies involving family caregivers have extensively linked the severity of caregiver burden to the severity of older patients’ cognitive impairment or dementia [11,12,13]. Furthermore, the progressively reduced independence and the occurrence of behavioral disturbances, such as irritability and aggression, have been suggested as additional sources of caregiver burden among family caregivers of older subjects with dementia [14,15,16].

The association between caregiver burden and the frailty of older adults has gained increasing interest in the last decade, as frailty has become included among age-related factors, which can negatively affect the caregiving experience. Frailty is defined as a state of increased vulnerability of the individual to stressors due to reduced homeostatic reserves, and it substantially results from the progressive decline of multiple physiological systems during the life course [17]. Two established approaches to evaluate frailty are represented by the phenotype model [18] and the deficit accumulation model [19]. The first essentially describes physical frailty and clusters the subjects as robust, pre-frail, and frail based on the evaluation of weight loss, fatigue, gait speed, handgrip strength, and sedentary habits. The model proposed by Rockwood and Mitnitski [19] alternatively describes multidimensional frailty based on the weight of different age-related deficits accumulated over time; typically, frailty is measured by calculating a Frailty Index (FI), which expresses the ratio between the deficits that an individual presents and the number of age-related health variables considered in the evaluation.

Previous evidence has mainly indicated that worse physical frailty in elderly patients contributes to an increased burden on family caregivers [20,21,22]. In the majority of these studies, frailty was measured according to the frailty phenotype model (or by the evaluation of physical proxies, such as gait speed and balance) or by using only partially multidimensional tools. Although the evidence is consistent with the negative impact of physical frailty on caregiver burden, a high heterogeneity in the tools used to assess burden has often emerged [20].

The interaction between the psychological features of older adults and caregiver burden is a stimulating topic of research, in line with an increasingly multifactorial approach to caregiver burden [23]. Several studies involving older adults with dementia have predominantly discussed the psychological factors of patients within the wide construct of behavioral and psychological symptoms in dementia (BPSD), highlighting the negative effect of patients’ depressive behaviors, apathy, or euphoria on caregiver wellbeing [24]. The impact of older patients’ depression on caregiver burden has been further confirmed, supporting the importance of an accurate evaluation of depression, which can benefit both patients with dementia and caregivers [25]. In addition, patients’ psychological factors, such as reduced spiritual wellbeing and health-related quality of life, have been suggested as further sources of burden among caregivers of older adults with Parkinson’s disease [26].

Dispositional optimism is defined as a stable dispositional factor reflecting an individual’s positive expectations for the future; furthermore, it is acknowledged as one of the psychological factors involved in adaptation to aging in older adults by promoting the adoption of healthy behaviors and a consequently better quality of life [27,28,29]. Previous studies have assessed dispositional optimism among caregivers of patients suffering from different medical conditions. The findings of these studies highlighted that caregivers’ levels of optimism might contribute to their mental wellbeing and distress [30,31,32]. To the best of our knowledge, the contribution of older patients’ dispositional optimism to caregiver burden has been poorly investigated.

In light of these premises, we first focused on exploring the level of burden among a sample of family caregivers of older adults. In addition, we aimed at investigating the interactions between the level of care-related burden and different clinical characteristics measured in older care recipients. Specifically, we aimed at investigating whether older care recipients’ multidimensional frailty and optimism might be differently associated with the burden of their family caregivers. In line with the mentioned previous evidence, we expected that even multidimensional frailty evaluated in older care recipients might significantly interact with the level of burden perceived by caregivers. Additionally, in line with its theoretical framework, we hypothesized that dispositional optimism might be a health-oriented psychological factor that is beneficial not only for older care recipients but also for their family caregivers.

## 2. Materials and Methods

### 2.1. Study Design and Participants

The present research is a cross-sectional study that involved a sample of family caregivers and their respective care recipients. The older adults involved in the study were outpatients referred to the Geriatrics and Multidimensional Evaluation Clinic (University of Messina, Italy); therefore, patients with an age ≥ 65 years were evaluated.

### 2.2. Procedure

Patients and caregivers were recruited on a voluntary basis during the first visits scheduled at the clinic. The patients usually undergo visits after an appointment; visits are generally suggested by general practitioners or sometimes spontaneously requested by patients. The recruitment took place from October 2018 to October 2019 and involved family members and patients who referred to the clinic for the first time.

The inclusion criterion for caregivers was that they effectively acted as caregivers for their elderly family members based on their relevant help and supervision in carrying out daily life activities. Specifically, we evaluated the effective contribution of caregivers in helping older care recipients to carry out basic and instrumental activities of daily life. For this purpose, we used the items contained in the Basic Autonomy in Daily Living (BADL) [33] and the Instrumental Autonomy of Daily Living (IADL) [34] scales as a source of information in order to qualify a family member as a caregiver.

The presence of severe major neurocognitive disorders [35] and the presence of severe functional limitations (e.g., inability to walk, severe limitations in the upper limbs, severe diagnosed sensory deficits) were considered exclusion criteria for patients. These criteria were justified by the need for patients to undergo an assessment of frailty based on multifactorial investigations, which included cognitive and physical functioning, among other variables (see Table S1 in the Supplementary Material for further details).

Each procedure completed in the present study was in accordance with the ethical standards of our institutional research committee and with the 1964 Helsinki Declaration and its later amendments. Informed consent was obtained from each participant. The Ethics Committee of the University Hospital of Messina approved the protocol of this study (Prot. 23/19).

### 2.3. Measures

The Caregiver Burden Inventory (CBI) was used to measure the multidimensional care-related burden of caregivers [36]. The CBI is a self-report, 24-item questionnaire with an answer system based on a five-point Likert scale ranging from 0 to 4 according to the frequency of perceived burden. High scores refer to an increased level of burden. The Italian version of the CBI was employed in this study, which showed a high internal consistency (Cronbach’s alpha value > 0.80) [37]. The questionnaire explores five different burden dimensions: time-dependence burden (CBI TD), developmental burden (CBI EV), physical burden (CBI PHYS), social burden (CBI SOC), and emotional burden (CBI EMOT). CBI TD refers to the burden caused by the restriction of the caregiver’s personal time, and this dimension evaluates the stress related to the time that the caregiver must devote to the care recipient. CBI EV refers to the sense of failure relative to the caregiver’s expectations and hopes. CBI PHYS is a measure of care-related physical stress and somatic disorders. CBI SOC is linked to conflicts in the caregiver’s personal and professional life. Finally, CBI EMOT refers to the caregiver’s feelings of embarrassment and shame caused by the patient’s behavior. Each dimension is evaluated by five items, except for the CBI TD, which is evaluated by four items; therefore, the score for each dimension ranges from 0 to 20, with the exception of the CBI TD, which, in order to be compared with the other dimensions, needs to be recalculated by multiplying the score by a correction factor of 1.25. The CBI also provides a total score of caregiver burden (CBI Total), with high scores representative of high levels of burden. The total score ranges from 0 to 100.

The frailty status of each patient was evaluated by the calculation of a 35-item Frailty Index (FI), according to the standard procedure [38]. The FI usually expresses the ratio of the number of deficits present to the number of total deficits considered; the higher the number of deficits detected, the more severe the frailty grade. Subjects with a FI ≥ 0.25 are commonly classified as frail [19]. The thirty-five variables that were checked for the calculation of the FI are reported in the Supplementary Materials (Table S1).

The revised Life Orientation Test (LOT-R) was used to evaluate the dispositional optimism of the patients [39]. We administered the Italian version of the LOT-R, which showed a Cronbach alpha of 0.81 [40]. The LOT-R is a 10-item questionnaire based on a 5-point Likert scale ranging from 1 (“strongly disagree”) to 5 (“strongly agree”). Items 1, 4, and 10 are phrased positively and oriented to optimism. Items 3, 7, and 9 are phrased negatively, denoting the inverse direction of optimism. Items 2, 5, 6, and 8 are considered distractors, and they are not scored. The score ranges from 0 to 24, and higher scores reflect a greater individual expectation of positive results.

### 2.4. Data Analysis

Data were analyzed using IBM SPSS 22 (IBM, Armonk, NY, USA) statistical software. We reported descriptive data in terms of the mean, Standard Deviation (SD), and percentage. The normal distribution of the investigated variables was verified by calculating skewness and kurtosis. Correlations were explored through Pearson’s coefficient.

We performed multivariate linear regressions to investigate the contribution of patients’ frailty status and optimism on caregiver burden; at the same time, we wanted to verify in what proportion the LOT-R could contribute to caregiver burden compared to the FI.

Values of *p* < 0.05 were considered statistically significant.

## 3. Results

### 3.1. Characteristics of the Participants

The study involved eighty family caregivers (mean age 64.28 ± 8.6) and eighty older patients (mean age 80.45 ± 7.13).

The caregivers were predominantly females; the majority of caregivers were sons or daughters (60% of the sample) and husbands or wives (approximately the 36% of the sample). The patients were predominantly females, and the majority of the patients were either married (approximately the 43% of the sample) or widowed (approximately the 47% of the sample). The main characteristics of caregivers and patients are summarized in Table 1.

### 3.2. Descriptive Statistics and Correlations

The average burden expressed by the caregivers did not reach a very high level, as expressed by the CBI Total score (16.32 ± 11.33). The higher score among caregiver burden dimensions was reached in the CBI TD (mean 7.66 ± 4.77). The patients exhibited a mean FI of 0.29; additionally, they exhibited a mean LOT-R score of 16.98.

We explored correlations between the following variables: patient’s age, the FI score, the LOT-R score, and the scores of the CBI. Significant positive correlations were found between each dimension of the CBI (including the CBI Total score) and the FI, which means that the caregivers of those patients with a worse frailty status exhibited higher levels of burden. Moreover, significant negative correlations were found between each dimension of the CBI (including the CBI Total score) and the score obtained on LOT-R by the patients; therefore, these correlations suggest that higher levels of burden found in the caregivers corresponded to lower levels of optimism in the care recipients.

Descriptive statistics and correlations are summarized in Table 2.

### 3.3. Multivariate Regression Models for Caregiver Burden Dimensions

We developed multivariate regression models for each caregiver burden dimension (i.e., CBI TD, CBI EV, CBI PHYS, CBI SOC, CBI EMOT) and for the CBI Total. The patient’s age was included only in the models for the CBI Total and CBI TD, in line with the previous correlation analysis.

The model for the CBI Total included the patient’s age and FI in the first step (R^2^ = 0.474; *p* < 0.001), and the LOT-R was included afterwards. The final step showed that the major significant contributions resulted from FI (β = 0.629) and LOT-R (β = −0.193), suggesting that higher frailty status and lower levels of optimism among patients significantly contributed to higher levels of overall burden among caregivers.

The model for the CBI TD similarly included the patient’s age and FI in the first step (R^2^ = 0.529; *p* < 0.001), and the LOT-R was included afterwards. The final step showed that the major significant contributions resulted from FI (β = 0.658) and LOT-R (β = −0.223), suggesting that higher frailty status and lower levels of optimism among patients significantly contributed to higher levels of burden caused by the restriction of personal time among caregivers.

The FI and the LOT-R were included in the models for each remaining burden dimension. According to the analyses, the FI was the only significant contributor to CBI EV (β = 0.528), CBI PHYS (β = 0.617), CBI SOC (β = 0.344), and CBI EMOT (β = 0.323). This evidence suggested that a worse frailty status among patients significantly contributed to a caregivers’ greater feelings of failure, physical stress, role conflicts, and embarrassment due to the patients’ behavior, respectively.

The final steps of the multivariate linear regression models are reported in Table 3.

## 4. Discussion

The purpose of the present study was to investigate whether frailty status and dispositional optimism, assessed in a sample of older adults, could contribute to the level of burden assessed among their family caregivers. Our findings showed that higher degrees of frailty and lower levels of optimism among older adults were associated with higher levels of overall burden and higher levels of time-dependent burden among caregivers. A worse frailty status among patients was additionally associated with higher levels of developmental, physical, social, and emotional burden among caregivers, as expressed by the remaining CBI scores.

The associations between the burden of caregivers and the severity of different age-related conditions among older adults have been previously explored [41,42,43]. In this context, the impact of older adults’ frailty status on caregiver burden has gained increasing interest in the last several years, highlighting that the degree of care recipients’ frailty should be considered a further source of burden for caregivers. Previous studies have predominantly focused on physical frailty in line with the frailty phenotype model or on the evaluation of surrogates of frailty, such as the daily autonomy status [20,44]. In studies involving caregivers and older adults, the adoption of a broader assessment of frailty still appears to be lacking and mostly limited to the use of tools that only partially capture the complexity of elderly subjects [22,45].

The present study involved a sample of older outpatients, so we needed to understand the complexity of their health status in order to investigate it as a source of burden for caregivers, rather than exclusively focusing on their physical frailty. Therefore, we evaluated the patients’ frailty through the FI, which is an expression of global health status, since it is calculated by accounting for variables of different nature (Table S1). Compared to a physical measure, the FI is a helpful strategy to capture outpatients’ clinical complexity, since it does not categorically define the presence or absence of a risk factor for subsequent age-related outcomes (e.g., disability), but it does represent a measure of the organism’s capacity to accumulate deficits that act together to reduce individual reserves [46]. Our findings confirm our preliminary hypothesis, suggesting that the burden of family caregivers may also be exacerbated by the complex frailty status of their care recipients, with a negative impact on a wide range of burden-related aspects, such as the caregiver’s restriction of personal time, feeling of failure, physical stress, perception of role conflicts, and feeling of embarrassment.

The findings of our study additionally showed the association of patients’ dispositional optimism with caregiver burden. According to the original definition, optimism is considered one of the two sides of an individual’s life orientation, and the opposite side is configured as pessimism; as a stable dispositional factor, optimism reflects the individual positive expectations for the future [47]. This optimistic drive translates into daily life in the adoption of proactive and health-oriented behaviors, with benefits in terms of both general wellbeing and physical health, especially in the presence of adversity to overcome [48,49]. In support of this conception, recent studies have confirmed that higher levels of optimism can allow patients with chronic diseases to face their difficulties more effectively and with more effort, with positive implications for disease treatment [50,51]; similarly, health workers with a more optimistic disposition frequently exhibit lower levels of emotion exhaustion [52].

The link between patients’ optimism and levels of caregiver burden has been poorly investigated, especially when patients are older adults. Previous evidence has suggested that lower levels of optimism among men who had to undergo coronary artery bypass surgery predicted higher levels of burden among their wives at 18-month follow-up [53]. The debate regarding how the levels of optimism exhibited by patients can affect the wellbeing of caregivers is very stimulating and important. In this context, caregivers may benefit from the adoption of health-oriented behaviors by optimistic patients, which in turn would ease the caregiver burden [54]. Our findings appear to be in line with these observations, supporting our hypothesis that patients’ dispositional optimism may be associated with the wellbeing of both patients and their caregivers.

We acknowledge that the study presents some limitations. First, the cross-sectional design does not allow for the making of causal inferences; therefore, longitudinal studies are strongly encouraged to better clarify the impact of patients’ optimism and frailty on caregiver burden. Moreover, the relatively small sample size and the predominant presence of female participants are additional limitations that should be overcome in future studies in order to better generalize the findings and to highlight potential gender differences. Additionally, a further limitation is represented by the age of the included caregivers (64.28 ± 8.6), which appears close to that of the care recipients. This evidence could affect the perception of care-related burden. Therefore, we strongly encourage researchers to include younger caregivers in future studies in order to better highlight potential age-related differences. Ultimately, we acknowledge the lack of caregiver-related adjustment factors, which might have enriched our analyses.

Understanding the close connection between patient-related factors and the burden of caregivers therefore appears to be an actual challenge with significant clinical, social, and public health implications. Caregivers of older care recipients, especially if they are family caregivers, play a delicate role that is often complicated by the various requests and care needs of the care recipients [55,56,57]. In this context, it is necessary to become increasingly aware not only of the difficulties of caring for frail elderly but also of the consequences on the wellbeing of the caregivers, who should not lack social and professional support [58,59]. Furthermore, caregivers should be routinely included in educational health-oriented programs and strategies, jointly with patients and care professionals, in order to improve their knowledge and the quality of care [60,61,62].

## 5. Conclusions

This study was based on the assessment of multidimensional frailty and levels of optimism in a sample of elderly outpatients and highlighted how these factors can be differently associated with the levels of burden expressed by their family caregivers.

The findings of the study support the idea that the frailty of elderly patients can represent a significant source of burden for family caregivers, especially if this frailty is an expression of the care recipients’ clinical complexity.

Several psychological factors are involved in the aging processes; in this context, the role of psychological contributors to aging adaptation is an interesting topic of research. Dispositional optimism is acknowledged as the ability to promote healthy behaviors, as well as the ability to favor a positive adaptation to aging. Patients’ positive expectations about their future might indirectly improve the caregiving experience, especially for family members.

Family caregivers are often a vital support for their elderly care recipients. Typically, it is increasingly necessary for them to be able to recognize the different alarm bells in the care recipients’ physical, functional, and psychological health in order to improve the quality of care. A possible suggestion for family caregivers is therefore to face the challenges of aging by leveraging the patient’s strengths and sharing their expectations and concerns about the future. The main effect of this joint perspective will be to improve the care recipients’ wellbeing and reduce the burden of caregivers.

Our findings could be considered a starting point in order to encourage further debate on the nature of this link.

## Figures and Tables

**Table 1:** Main characteristics of the participants.

	Family Caregivers (N = 80)	Patients (N = 80)
Age (mean ± SD)	64.28 ± 8.6	80.45 ± 7.13
Education (mean ± SD)	10.9 ± 6.5	6.35 ± 3.52
Gender male (%)	37.6	33.5
Gender female (%)	62.4	66.5
Married (%)	-	43.9
Widowed (%)	-	47.6
Other (%)	-	8.5
*Kinship with the patient*		
Son/Daughter (%)	60	-
Husband/Wife (%)	36.2	-
Other family member (%)	3.8	-
*Clinical characteristics*		
Frail subjects (%)	-	61.8
Not frail subjects (%)	-	38.2

SD = Standard Deviation.

**Table 2:** Descriptive statistics and Pearson’s correlations between variables.

Variables	Mean (± SD)	1	2	3	4	5	6	7	8
1. Patient’s age	80.45 ± 7.13								
2. FI	0.29 ± 0.09	0.198 *							
3. LOT-R	16.98 ± 5.38	ns	−0.329 **						
4. CBI TD	7.66 ± 4.77	0.172 *	0.750 **	−0.439 **					
5. CBI EV	3.54 ± 3.64	ns	0.638 **	−0.312 **	0.794 **				
6. CBI PHYS	3.01 ± 2.1	ns	0.651 **	−0.345 **	0.786 **	0.776 **			
7. CBI SOC	1.42 ± 1.91	ns	0.387 **	−0.288 **	0.547 **	0.724 **	0.722 **		
8. CBI EMOT	0.70 ± 1.12	ns	0.352 **	−0.230 **	0.538 **	0.693 **	0.680 **	0.819 **	
9. CBI Total	16.32 ± 11.33	0.176 *	0.705 **	−0.400 **	0.914 **	0.924 **	0.916 **	0.790 **	0.758 **

* = significant at *p* < 0.05; ** = significant at *p* < 0.01; ns = not significant.

**Table 3:** Multivariate linear regressions for the caregiver burden dimensions.

Dependent Variables	Tested Variables	Model				Coefficients			
		**R^2^**	**R^2^ Adjusted**	**F**	***p***	**SE(B)**	**β**	**t**	***p***
CBI Total		0.507	0.490	28.85	**<0.001**				
	Patient’s age					0.134	−0.048	−0.618	0.53
	FI					9.79	0.629	7.69	**<0.001**
	LOT-R					0.167	−0.193	−2.38	**0.019**
CBI TD		0.573	0.558	37.62	**<0.001**				
	Patient’s age					0.048	−0.077	−1.06	0.28
	FI					3.48	0.658	8.65	**<0.001**
	LOT-R					0.059	−0.223	−2.96	**0.004**
CBI EV		0.346	0.331	22.52	**<0.001**				
	FI					2.88	0.528	5.68	**<0.001**
	LOT-R					0.049	−0.138	−1.49	0.14
CBI PHYS		0.458	0.446	35.97	**<0.001**				
	FI					2.64	0.617	7.30	**<0.001**
	LOT-R					0.045	−0.141	−1.67	0.09
CBI SOC		0.189	0.169	9.87	**<0.001**				
	FI					1.73	0.344	3.32	**0.001**
	LOT-R					0.030	−0.175	−1.69	0.09
CBI EMOT		0.146	0.126	7.25	**0.001**				
	FI					1.12	0.323	3.04	**0.003**
	LOT-R					0.019	−0.124	−1.16	0.24

Significant *p* values are in bold.

## Data Availability

The raw data supporting the conclusions of this article will be made available on request by the corresponding author, without undue reservation.

## Supplementary Materials

The following are available online at https://www.mdpi.com/1660-4601/18/7/3406/s1. Table S1: Variables considered in the calculation of the Frailty Index (FI).
