# Supplementary material for: A Clinical Bridge between Family Caregivers and Older Adults: The Contribution of Patients’ Frailty and Optimism on Caregiver Burden
Source: Int J Environ Res Public Health. 2021 Mar 25;18(7):3406. doi: 10.3390/ijerph18073406 (PMC8037634; doi:10.3390/ijerph18073406)
Supplement: Supplementary file 1 [file ijerph-18-03406-s001.pdf]

Table S1. Variables considered in the calculation of the Frailty Index (FI)

Hospitalization	Pain	Urinary incontinence	Heart failure	Cerebrovascular disease
Fractures	Bathing	Faecal incontinence	Chronic Obstructive Pulmonary Disease	Handgrip strength
Caregiver	Dressing	Telephone	Body Mass Index	Parkinsonism
Cognitive status	Walking	Drugs	Cancer	Gait speed
Malnutrition	Getting up / Sitting down	Hypertension	Cirrhosis	Medications
Dehydration	Feeding	Diabetes	Chronic kidney failure	Benzodiazepines
Oral health	Toileting	Heart disease	Obesity	Neuroleptics
			**Total number of detected deficits** ___ / 35	**Frailty Index** ______

Note: The FI is expressed as a ratio of health deficits present to the total number of deficits considered; the greater the number of health deficits, the higher the degree of frailty. According to this approach, patients with a FI ≥0.25 are commonly considered frail.
